# *In Vitro* Susceptibility of Gram-Negative Pathogens to Cefiderocol in Five Consecutive Annual Multinational SIDERO-WT Surveillance Studies, 2014 to 2019

**DOI:** 10.1128/AAC.01990-21

**Published:** 2022-02-15

**Authors:** James A. Karlowsky, Meredith A. Hackel, Miki Takemura, Yoshinori Yamano, Roger Echols, Daniel F. Sahm

**Affiliations:** a IHMA, Schaumburg, Illinois, USA; b Department of Medical Microbiology and Infectious Diseases, Max Rady College of Medicine, University of Manitoba, Winnipeg, Manitoba, Canada; c Laboratory for Drug Discovery and Disease Research, Shionogi & Co., Ltd., Osaka, Japan; d Research Planning Department, Shionogi & Co., Ltd., Osaka, Japan; e Infectious Disease Drug Development Consulting, LLC, Easton, Connecticut, USA

**Keywords:** cefiderocol, Gram-negative bacilli, CRE, carbapenem-resistant *Enterobacterales*, *Pseudomonas aeruginosa*, *Acinetobacter baumannii*, *Stenotrophomonas maltophilia*

## Abstract

We report *in vitro* susceptibility data from five consecutive annual SIDERO-WT surveillance studies (2014 to 2019) for cefiderocol and comparators tested against Gram-negative clinical isolates from North America and Europe. CLSI broth microdilution was used to determine MICs for *Enterobacterales* (*n *= 31,896), Pseudomonas aeruginosa (*n *= 7,700), Acinetobacter baumannii complex (*n *= 5,225), Stenotrophomonas maltophilia (*n *= 2,030), and Burkholderia cepacia complex (*n *= 425). MICs were interpreted by CLSI-approved clinical breakpoints (February 2021). Cefiderocol inhibited 99.8, 96.7, 91.6, and 97.7% of all *Enterobacterales*, meropenem-nonsusceptible, ceftazidime-avibactam-nonsusceptible, and ceftolozane-tazobactam-nonsusceptible isolates, respectively, at ≤4 μg/mL (susceptible breakpoint). Cefiderocol inhibited 99.9, 99.8, 100, and 99.8% of all P. aeruginosa, meropenem-nonsusceptible, ceftazidime-avibactam-nonsusceptible, and ceftolozane-tazobactam-nonsusceptible isolates, respectively, at ≤4 μg/mL (susceptible breakpoint). Cefiderocol inhibited 96.0% of all A. baumannii complex isolates and 94.2% of meropenem-nonsusceptible isolates at ≤4 μg/mL (susceptible breakpoint) and 98.6% of S. maltophilia isolates at ≤1 μg/mL (susceptible breakpoint). B. cepacia complex isolates were tested with a MIC_50_ of ≤0.03 μg/mL and MIC_90_ of 0.5 μg/mL. Annual cefiderocol percent susceptible rates for *Enterobacterales* (North America range, 99.6 to 100%/year; Europe range, 99.3 to 99.9%/year) and P. aeruginosa (North America range, 99.8 to 100%; Europe range, 99.9 to 100%) were unchanged from 2014 to 2019. Annual percent susceptible rates for A. baumannii complex demonstrated sporadic, nondirectional differences (North America range, 97.5 to 100%; Europe range, 90.4 to 97.5%); the wider range for Europe (∼7%) was due to isolates from Russia. Annual percent susceptible rates for S. maltophilia showed minor, nondirectional differences (North America range, 96.4 to 100%; Europe range, 95.6 to 100%). We conclude that clinical isolates of *Enterobacterales* (99.8% susceptible), P. aeruginosa (99.9%), A. baumannii (96.0%), and S. maltophilia (98.6%) collected in North America and Europe from 2014 to 2019 were highly susceptible to cefiderocol.

## INTRODUCTION

The prevalence of carbapenem-resistant, multidrug-resistant (MDR), and difficult-to-treat Gram-negative bacilli is increasing worldwide, and therapeutic options for infected patients are often limited (1–3). The World Health Organization (WHO) has classified carbapenem-resistant *Enterobacterales*, Pseudomonas aeruginosa, and Acinetobacter baumannii as pathogens of the highest (critical) priority for development of new antibacterial agents (2). Cefiderocol, a parenteral siderophore cephalosporin, was approved by the United States Food and Drug Administration (FDA) in November 2019 for the treatment of adults with complicated urinary tract infections, including pyelonephritis, caused by susceptible Gram-negative bacilli (Escherichia coli, Klebsiella pneumoniae, Proteus mirabilis, Enterobacter cloacae complex, and P. aeruginosa) when limited or no other treatment options exist (4). In April 2020, the European Medicines Agency (EMA) licensed cefiderocol for the treatment of infections due to aerobic Gram-negative organisms in adults with limited treatment options (5). In September 2020, the FDA approved cefiderocol for a new indication, the treatment of hospital-acquired bacterial pneumonia and ventilator-associated bacterial pneumonia caused by *Enterobacterales* (E. coli, K. pneumoniae, E. cloacae complex, and Serratia marcescens), P. aeruginosa, and Acinetobacter baumannii complex. Clinical development of cefiderocol continues for the treatment of serious infections attributable to resistant Gram-negative bacilli, including infections caused by carbapenem-resistant Gram-negative bacilli (6).

Cefiderocol possesses a unique mechanism of bacterial cell entry, making it an important addition to the antimicrobial armamentarium. The optimized chloro-catechol moiety within the C-3 side chain of cefiderocol facilitates formation of chelated complexes with ferric iron and expedites its transport across the outer membrane of Gram-negative bacilli using constitutive iron transport systems (7). Following its delivery to the periplasmic space, cefiderocol binds primarily to penicillin binding protein 3 (PBP 3), similarly to other cephalosporins, and impedes peptidoglycan synthesis (7). Cefiderocol has been shown to be stable to hydrolysis by most clinically important β-lactamases, including both serine β-lactamases of Ambler classes A (e.g., KPC and, extended-spectrum lactamase [ESBL; e.g., CTX type, SHV type, and TEM type]), C (i.e., AmpC), and D (e.g., OXA) carbapenemases and metallo-β-lactamases of Ambler class B (e.g., IMP, NDM, and VIM) and to be minimally affected by porin deletions and efflux-mediated resistance mechanisms (2- to 4-fold increases in cefiderocol MIC) (7–14).

Using current standardized reference testing methods and reliable, predictable, evidence-driven MIC and disk diffusion zone size interpretative criteria to determine *in vitro* activities for recently approved and investigational agents is critical to establishing and supporting treatment decisions and expanding the role of these agents in patient care, particularly for patients where unmet medical need exists (2). Investigational MIC and disk diffusion zone diameter interpretative criteria for cefiderocol were published by the Clinical and Laboratory Standards Institute (CLSI) in 2019 based on *in vitro* activity and preclinical *in vivo* pharmacokinetic/pharmacodynamics data prior to FDA approval of cefiderocol (15, 16). In February 2021, CLSI approved MIC clinical breakpoints for *Enterobacterales*, P. aeruginosa, and Acinetobacter species of ≤4 μg/mL (susceptible), 8 μg/mL (intermediate), and ≥16 μg/mL (resistant) and for Stenotrophomonas maltophilia of ≤1 μg/mL (susceptible) and >1 μg/mL (nonsusceptible) (17). The updated MIC clinical breakpoints for cefiderocol will be published in early 2022 with the release of the 32nd edition of the CLSI M100 document. Clinical breakpoints for cefiderocol are also available from the United States Food and Drug Administration (FDA) (18) and the European Committee on Antimicrobial Susceptibility Testing (EUCAST) (19) (see Table S1 in the supplemental material) but were not used for MIC interpretation in this article.

The intent of the current study was to evaluate the *in vitro* susceptibility to cefiderocol of Gram-negative pathogens (*Enterobacterales*, P. aeruginosa, A. baumannii complex, S. maltophilia, and Burkholderia cepacia complex) collected over five consecutive annual SIDERO-WT surveillance studies (from November 2014 to December 2019) conducted in North America and Europe using the recently approved (February 2021) CLSI MIC clinical breakpoints (17). In addition, we performed cefiderocol susceptibility subset analysis that included isolates with meropenem-, ceftazidime-avibactam-, and ceftolozane-tazobactam-nonsusceptible phenotypes, as it is in patients infected with these nonsusceptible isolates where cefiderocol use most directly addresses an unmet medical need, and reviewed the cefiderocol percent susceptible rates and isolate MIC distributions by year to identify trends in cefiderocol *in vitro* activity over time.

## RESULTS

The minimal inhibitory concentrations of cefiderocol that inhibited 50% (MIC_50_) and 90% (MIC_90_) of the 31,896 isolates of *Enterobacterales* tested from North America and Europe from 2014 to 2019 were 0.12 and 1 μg/mL, respectively (Table 1). Cefiderocol inhibited 99.8% of all isolates of *Enterobacterales* at a MIC of ≤4 μg/mL. The cefiderocol MIC_50_ and MIC_90_ were 1 and 4 μg/mL, respectively, for the subset of 1,021 meropenem-nonsusceptible (MIC, ≥2 μg/mL) isolates of *Enterobacterales*; 96.7% of meropenem-nonsusceptible isolates were susceptible to cefiderocol. Cefiderocol demonstrated a higher percent susceptible rate against meropenem-nonsusceptible isolates (≥20% higher) than ceftazidime-avibactam (77.0%), cefepime (8.7%), ceftolozane-tazobactam (7.8%), and ciprofloxacin (7.8%). A total of 91.6% of 263 isolates of ceftazidime-avibactam-nonsusceptible (MIC, ≥16 μg/mL) *Enterobacterales* and 97.7% of 2,658 isolates of ceftolozane-tazobactam-nonsusceptible (MIC, ≥4 μg/mL) *Enterobacterales* were susceptible to cefiderocol. In comparison, only 3.8% of ceftazidime-avibactam-nonsusceptible *Enterobacterales* isolates were susceptible to ceftolozane-tazobactam and 90.5% of ceftolozane-tazobactam-nonsusceptible *Enterobacterales* isolates were susceptible to ceftazidime-avibactam. MIC_90_ values for colistin (excluding isolates with intrinsic resistance—Proteus spp., *Providencia* spp., Morganella morganii, and S. marcescens) and ciprofloxacin were 1 and >8 μg/mL, respectively, for all isolates of *Enterobacterales* tested.

**TABLE 1:** Cumulative antimicrobial susceptibility testing results from SIDERO-WT surveillance study isolates of *Enterobacterales*, P. aeruginosa, A. baumannii complex, S. maltophilia, and B. cepacia complex collected in North America and Europe from 2014 to 2019

Organism/phenotype (no. of isolates)	Antimicrobial agent(s)	MIC (μg/mL)			CLSI MIC interpretation^*a*^		
		Range	MIC_50_	MIC_90_	% susceptible	% intermediate	% resistant
*Enterobacterales* ^*b*^							
All isolates (31,896)	Cefiderocol	≤0.002 to >256	0.12	1	99.8	0.2	0.1
	Cefepime	≤0.06 to >64	≤0.12	16	85.9	3.0	11.2
	Ceftazidime-avibactam	≤0.03 to >64	0.12	0.5	99.2	NA	0.8
	Ceftolozane-tazobactam	≤0.06 to >64	0.25	2	91.7	1.8	6.6
	Ciprofloxacin	≤0.06 to >8	≤0.12	>8	74.5	3.2	22.3
	Colistin	≤0.12 to >8	0.5	1	NA	96.8	3.2
	Meropenem	≤0.06 to >64	≤0.06	0.12	96.8	0.4	2.9

Meropenem nonsusceptible (MIC, ≥2 μg/mL) (1,021)	Cefiderocol	0.004 to >256	1	4	96.7	2.6	0.8
	Cefepime	≤0.06 to >64	64	>64	8.7	6.9	84.4
	Ceftazidime-avibactam	≤0.06 to >64	1	>64	77.0	NA	23.0
	Ceftolozane-tazobactam	0.25 to >64	64	>64	7.8	2.9	89.2
	Ciprofloxacin	≤0.06 to >8	>8	>8	7.8	2.9	89.2
	Colistin	≤0.12 to >8	0.5	>8	NA	80.5	19.6
	Meropenem	2 to >64	16	>64	0	10.9	89.1

Ceftazidime-avibactam nonsusceptible (MIC, ≥16 μg/mL) (263)	Cefiderocol	0.015 to >256	2	4	91.6	5.3	3.0
	Cefepime	≤0.06 to >64	64	>64	3.4	5.7	90.9
	Ceftazidime-avibactam	16 to >64	>64	>64	0	NA	100
	Ceftolozane-tazobactam	0.12 to >64	>64	>64	3.8	0.4	95.8
	Ciprofloxacin	≤0.06 to >8	>8	>8	7.2	5.7	87.1
	Colistin	≤0.12 to >8	0.5	2	NA	90.9	9.1
	Meropenem	≤0.06 to >64	>16	>64	10.7	3.4	85.9

Ceftolozane-tazobactam nonsusceptible (MIC, ≥4 μg/mL) (2,658)	Cefiderocol	0.004 to >256	1	4	97.7	1.7	0.6
	Cefepime	≤0.06 to >64	16	>64	30.7	14.6	54.7
	Ceftazidime-avibactam	≤0.06 to >64	1	8	90.5	NA	9.5
	Ceftolozane-tazobactam	4 to >64	16	>64	0	21.3	78.7
	Ciprofloxacin	≤0.06 to >8	8	>8	33.8	3.7	62.5
	Colistin	≤0.12 to >8	0.5	8	NA	88.7	11.3
	Meropenem	≤0.06 to >64	0.12	32	64.6	3.3	32.1


P. aeruginosa							
All isolates (7,700)	Cefiderocol	≤0.002 to 8	0.12	0.5	99.9	0.1	0
	Cefepime	≤0.06 to >64	4	16	82.9	9.1	8.0
	Ceftazidime-avibactam	≤0.03 to >64	2	8	93.8	NA	6.2
	Ceftolozane-tazobactam	≤0.06 to >64	0.5	2	94.0	1.0	5.0
	Ciprofloxacin	≤0.06 to >8	0.25	>8	70.8	6.5	22.7
	Colistin	≤0.12 to >8	1	2	NA	99.3	0.7
	Meropenem	≤0.06 to >64	0.5	16	77.2	5.8	17.0

Meropenem nonsusceptible (MIC, ≥4 μg/mL) (1,759)	Cefiderocol	≤0.002 to 8	0.25	1	99.8	0.2	0
	Cefepime	≤0.06 to >64	16	32	49.0	22.5	28.5
	Ceftazidime-avibactam	≤0.06 to >64	4	32	75.0	NA	25.0
	Ceftolozane-tazobactam	0.25 to >64	1	>64	76.1	3.3	20.6
	Ciprofloxacin	≤0.06 to >8	4	>8	31.2	9.4	59.4
	Colistin	≤0.12 to >8	1	1	NA	98.5	1.5
	Meropenem	4 to >64	8	64	0	25.5	74.5
							
Ceftazidime-avibactam nonsusceptible (MIC, ≥16 μg/mL) (477)	Cefiderocol	≤0.002 to 4	0.25	2	100	0	0
	Cefepime	1 to >64	32	>64	5.5	20.8	73.8
	Ceftazidime-avibactam	16 to >64	32	>64	0	NA	100
	Ceftolozane-tazobactam	0.5 to >64	64	>64	24.3	6.5	69.2
	Ciprofloxacin	≤0.06 to >8	>8	>8	10.7	5.5	83.9
	Colistin	≤0.12 to >8	1	2	NA	99.2	0.8
	Meropenem	0.12 to >64	32	>64	7.8	4.2	88.1
Ceftolozane-tazobactam nonsusceptible (MIC, ≥8 μg/mL) (463)	Cefiderocol	0.004 to 8	0.25	2	99.8	0.2	0
	Cefepime	1 to >64	32	>64	9.1	18.4	72.6
	Ceftazidime-avibactam	0.5 to >64	32	>64	22.0	NA	78.0
	Ceftolozane-tazobactam	8 to >64	64	>64	0	16.9	83.2
	Ciprofloxacin	≤0.06 to >8	>8	>8	7.8	5.0	87.3
	Colistin	≤0.12 to >8	1	2	NA	98.1	1.9
	Meropenem	≤0.06 to >64	>16	>64	9.1	5.4	85.5


A. baumannii complex							
All isolates (5,225)	Cefiderocol	≤0.002 to >256	0.12	1	96.0	1.3	2.7
	Cefepime	≤0.06 to >64	8	>64	52.0	9.5	38.5
	Ceftazidime-avibactam	≤0.06 to >64	16	>64	NA	NA	NA
	Ceftolozane-tazobactam	≤0.06 to >64	8	>64	NA	NA	NA
	Ciprofloxacin	≤0.12 to >8	>8	>8	40.0	0.7	59.3
	Colistin	≤0.25 to >8	0.5	2	NA	92.7	7.3
	Meropenem	≤0.06 to >64	16	>64	46.2	1.3	52.5

Meropenem nonsusceptible (MIC, ≥4 μg/mL) (2,810)	Cefiderocol	≤0.002 to >256	0.25	2	94.2	2.1	3.7
	Cefepime	≤0.12 to >64	32	>64	19.5	13.0	67.5
	Ceftazidime-avibactam	1 to >64	32	>64	NA	NA	NA
	Ceftolozane-tazobactam	≤0.06 to >64	16	>64	NA	NA	NA
	Ciprofloxacin	≤0.12 to >8	>8	>8	1.4	0.1	98.6
	Colistin	≤0.25 to >8	0.5	>8	NA	87.2	12.8
	Meropenem	4 to >64	64	>64	0.0	2.4	97.6


S. maltophilia							
All isolates (2,030)	Cefiderocol	≤0.002 to 128	0.06	0.25	98.6	NA	NA
	Cefepime	≤0.06 to >64	32	64	NA	NA	NA
	Ceftazidime-avibactam	≤0.06 to >64	16	64	NA	NA	NA
	Ceftolozane-tazobactam	≤0.06 to >64	16	>64	NA	NA	NA
	Ciprofloxacin	≤0.12 to >8	2	>8	NA	NA	NA
	Colistin	≤0.25 to >8	1	>8	NA	NA	NA
	Meropenem	≤0.06 to >64	>64	>64	NA	NA	NA


B. cepacia complex							
All isolates (425)	Cefiderocol	≤0.002 to 128	≤0.03	0.5	NA	NA	NA
	Cefepime	≤0.06 to >64	8	>64	NA	NA	NA
	Ceftazidime-avibactam	0.25 to >64	4	8	NA	NA	NA
	Ceftolozane-tazobactam	0.25 to >64	2	32	NA	NA	NA
	Ciprofloxacin	≤0.12 to >8	1	>8	NA	NA	NA
	Colistin	≤0.25 to >8	>8	>8	NA	NA	NA
	Meropenem	0.12 to 32	4	16	56.7	26.6	16.7

Meropenem nonsusceptible (MIC, ≥8 μg/mL) (184)	Cefiderocol	≤0.002 to 128	≤0.03	1	NA	NA	NA
	Cefepime	≤0.06 to >64	0.25	>64	NA	NA	NA
	Ceftazidime-avibactam	0.25 to >64	4	16	NA	NA	NA
	Ceftolozane-tazobactam	0.5 to >64	4	>64	NA	NA	NA
	Ciprofloxacin	≤0.12 to >8	2	>8	NA	NA	NA
	Colistin	≤0.25 to >8	>8	>8	NA	NA	NA
	Meropenem	8 to 32	8	>16	0	61.4	38.6

a Cefiderocol MICs and MICs for other antimicrobial agents were interpreted by CLSI breakpoints. CLSI MIC breakpoints for cefiderocol tested against *Enterobacterales* are as follows: susceptible, ≤4 μg/mL; intermediate, 8 μg/mL; and resistant, ≥16 μg/mL. CLSI MIC breakpoints for cefiderocol tested against P. aeruginosa are as follows: susceptible, ≤4 μg/mL; intermediate, 8 μg/mL; and resistant, ≥16 μg/mL. CLSI MIC breakpoints for cefiderocol tested against Acinetobacter spp. are as follows: susceptible, ≤4 μg/mL; intermediate, 8 μg/mL; and resistant, ≥16 μg/mL. CLSI MIC breakpoints for cefiderocol tested against S. maltophilia are as follows: susceptible, ≤1 μg/mL; and nonsusceptible, >1 μg/mL. CLSI currently does not publish cefiderocol MIC breakpoints for B. cepacia complex. For cefepime tested against *Enterobacterales* with MICs interpreted using CLSI breakpoints, susceptible dose-dependent isolates were classified as intermediate. NA, not available because MIC interpretative criteria are currently not available for this organism-antimicrobial agent combination.

b For *Enterobacterales* tested against colistin, isolates with intrinsic resistance to colistin (Proteus spp., *Providencia* spp., Morganella morganii, and Serratia marcescens) were excluded from calculating MIC and CLSI MIC interpretation results for all isolates (*n *= 24,388), meropenem-nonsusceptible isolates (*n *= 931), ceftazidime-avibactam-nonsusceptible isolates (*n *= 230), and ceftolozane-tazobactam-nonsusceptible isolates (*n *= 2,448).

**FIG 1:**
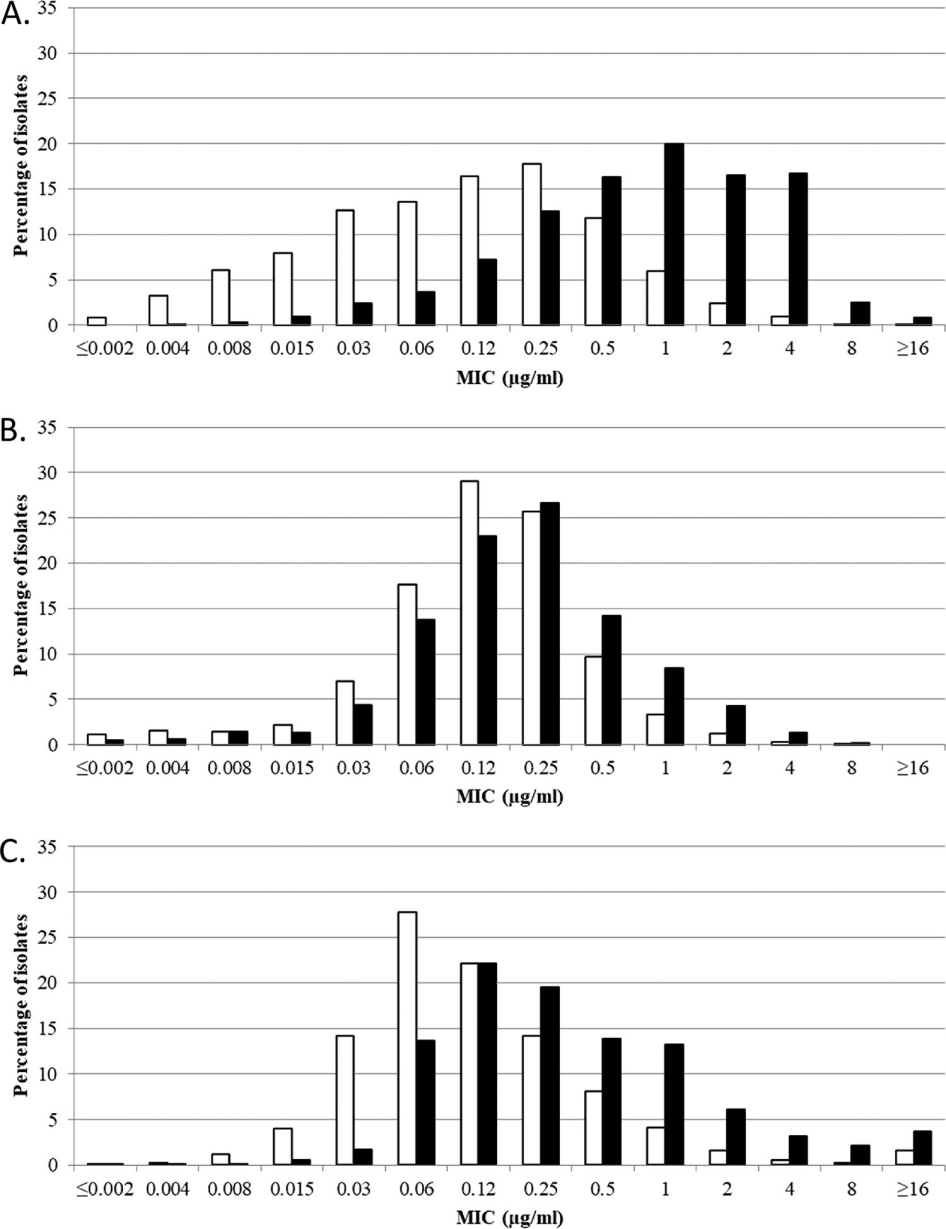
Cefiderocol MIC distributions for combined North America and Europe isolates of (A) meropenem-susceptible (MIC, ≤1 μg/mL) (white bars; *n *= 30,875) and meropenem-nonsusceptible (MIC, ≥2 μg/mL) (black bars; *n *= 1,021) *Enterobacterales*, (B) meropenem-susceptible (MIC, ≤2 μg/mL) (white bars; *n *= 5,941) and meropenem-nonsusceptible (MIC, ≥4 μg/mL) (black bars; *n *= 1,759) P. aeruginosa, and (C) meropenem-susceptible (MIC, ≤2 μg/mL) (white bars; *n *= 2,415) and meropenem-nonsusceptible (MIC, ≥4 μg/mL) (black bars; *n *= 2,810) A. baumannii complex. MIC breakpoints for cefiderocol tested against *Enterobacterales* are as follows: CLSI and FDA, susceptible, ≤4 μg/mL, intermediate, 8 μg/mL, and resistant, ≥16 μg/mL; EUCAST, susceptible, ≤2 μg/mL, and resistant, >2 μg/mL. MIC breakpoints for cefiderocol tested against P. aeruginosa are as follows: CLSI, susceptible, ≤4 μg/mL, intermediate, 8 μg/mL, and resistant, ≥16 μg/mL; FDA, susceptible, ≤1 μg/mL, intermediate, 2 μg/mL, and resistant, ≥4 μg/mL; EUCAST, susceptible, ≤2 μg/mL, and resistant, >2 μg/mL. MIC breakpoints for cefiderocol tested against A. baumannii complex are as follows: CLSI, susceptible, ≤4 μg/mL, intermediate, 8 μg/mL, and resistant, ≥16 μg/mL; EUCAST, susceptible, ≤2 μg/mL, and resistant >2 μg/mL. FDA does not publish MIC breakpoints for cefiderocol tested against isolates of Acinetobacter spp.

**FIG 2:**
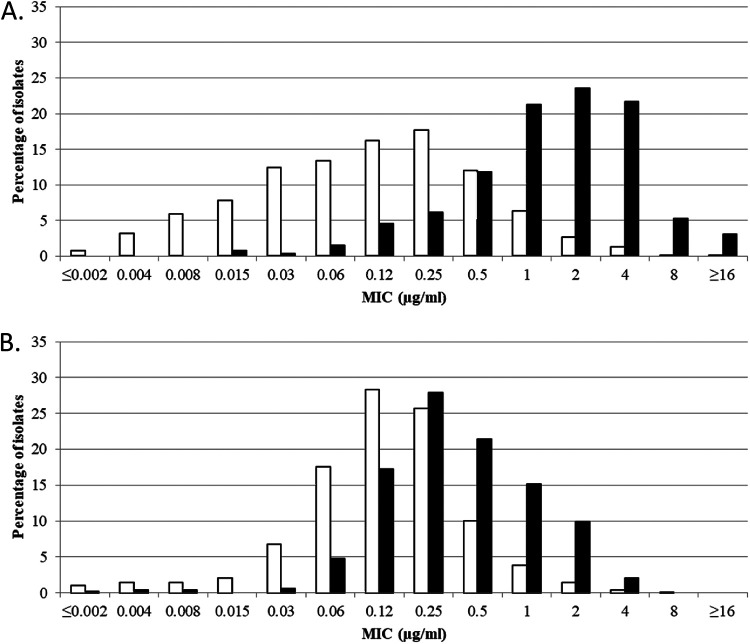
Cefiderocol MIC distributions for combined North America and Europe isolates of (A) ceftazidime-avibactam-susceptible (MIC, ≤8 μg/mL) (white bars; *n *= 31,633) and ceftazidime-avibactam-resistant (MIC, ≥16 μg/mL) (black bars; *n *= 263) *Enterobacterales* and (B) ceftazidime-avibactam-susceptible (MIC ≤8 μg/mL) (white bars; *n *= 7,223) and ceftazidime-avibactam-resistant (MIC, ≥16 μg/mL) (black bars; *n *= 477) P. aeruginosa. MIC breakpoints for cefiderocol tested against *Enterobacterales* are as follows: CLSI and FDA, susceptible, ≤4 μg/mL, intermediate, 8 μg/mL, and resistant, ≥16 μg/mL; EUCAST, susceptible, ≤2 μg/mL, and resistant >2 μg/mL. MIC breakpoints for cefiderocol tested against P. aeruginosa are as follows: CLSI, susceptible, ≤4 μg/mL, intermediate, 8 μg/mL, and resistant, ≥16 μg/mL; FDA, susceptible, ≤1 μg/mL, intermediate, 2 μg/mL, and resistant, ≥4 μg/mL; EUCAST, susceptible, ≤2 μg/mL, and resistant, >2 μg/mL.

**FIG 3:**
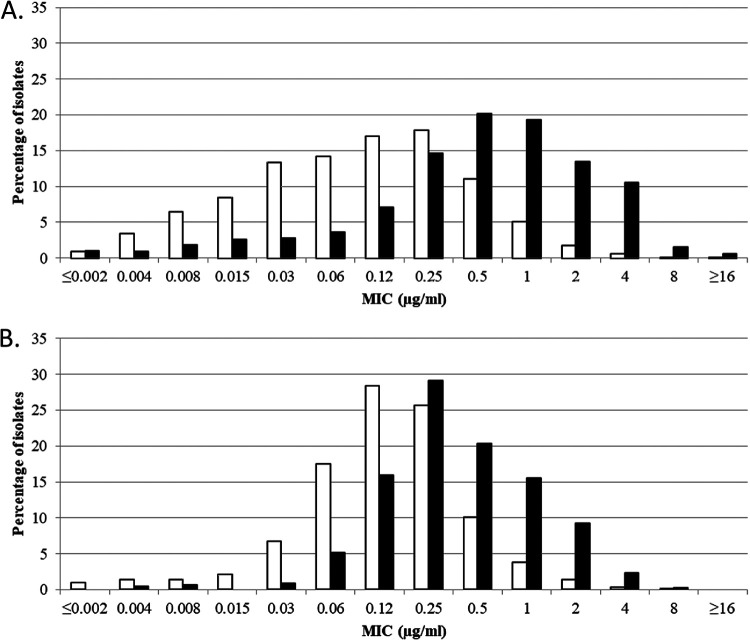
Cefiderocol MIC distributions for combined North America and Europe isolates of (A) ceftolozane-tazobactam-susceptible (MIC, ≤2 μg/mL) (white bars; *n *= 29,238) and ceftolozane-tazobactam-resistant (MIC, ≥4 μg/mL) (black bars; *n *= 2,950) *Enterobacterales* and (B) ceftolozane-tazobactam-susceptible (MIC, ≤4 μg/mL) (white bars; *n *= 7,237) and ceftolozane-tazobactam-resistant (MIC, ≥8 μg/mL) (black bars; *n *= 463) P. aeruginosa. MIC breakpoints for cefiderocol tested against *Enterobacterales* are as follows: CLSI and FDA, susceptible, ≤4 μg/mL, intermediate, 8 μg/mL, and resistant, ≥16 μg/mL; EUCAST, susceptible, ≤2 μg/mL, and resistant, >2 μg/mL. MIC breakpoints for cefiderocol tested against P. aeruginosa are as follows: CLSI, susceptible, ≤4 μg/mL, intermediate, 8 μg/mL, and resistant, ≥16 μg/mL; FDA, susceptible, ≤1 μg/mL, intermediate, 2 μg/mL, and resistant, ≥4 μg/mL; EUCAST, susceptible, ≤2 μg/mL, and resistant, >2 μg/mL.

Cefiderocol MICs for meropenem-nonsusceptible (Fig. 1), ceftazidime-avibactam-nonsusceptible (Fig. 2), and ceftolozane-tazobactam-nonsusceptible (Fig. 3) isolates of *Enterobacterales* (both North American and European isolates combined) demonstrated a rightward shift (of 1 to 3 doubling dilutions) to higher cefiderocol MICs compared to each respective antimicrobial-susceptible subset; however, as mentioned earlier, most meropenem (96.7%)-, ceftazidime-avibactam (91.6%)-, and ceftolozane-tazobactam (97.7%)-nonsusceptible isolates remained susceptible to cefiderocol, with MICs of ≤4 μg/mL.

The cefiderocol MIC_50_ and MIC_90_ were 0.12 and 0.5 μg/mL for 7,700 isolates of P. aeruginosa collected in North America and Europe from 2014 to 2019 (Table 1). Cefiderocol inhibited 99.9% of isolates at ≤4 μg/mL. Ceftazidime-avibactam (93.8% susceptible) and ceftolozane-tazobactam (94.0% susceptible) were less active than cefiderocol against all isolates of P. aeruginosa tested. The MIC_50_ and MIC_90_ values for cefiderocol against the subset of 1,759 isolates of meropenem-nonsusceptible (MIC, ≥4 μg/mL) P. aeruginosa were 0.25 and 1 μg/mL, respectively, and 99.9% of meropenem-nonsusceptible isolates were susceptible to cefiderocol. Ceftazidime-avibactam, ceftolozane-tazobactam, and cefepime were all tested with MIC_90_ values of 32 or >64 μg/mL against isolates of meropenem-nonsusceptible P. aeruginosa and exhibited percent susceptible rates of 76.1% (ceftolozane-tazobactam), 75.0% (ceftazidime-avibactam), and 49.0% (cefepime). MIC_90_ values for ciprofloxacin (31.2% susceptible) and colistin were >8 μg/mL and 1 μg/mL, respectively, for meropenem-nonsusceptible P. aeruginosa. A total of 100% of 477 isolates of ceftazidime-avibactam-nonsusceptible (MIC, ≥16 μg/mL) and 99.8% of 463 isolates of ceftolozane-tazobactam-nonsusceptible (MIC, ≥8 μg/mL) P. aeruginosa, respectively, were susceptible to cefiderocol. In comparison, only 24.3% of ceftazidime-avibactam-nonsusceptible P. aeruginosa isolates were susceptible to ceftolozane-tazobactam, and only 22.0% of ceftolozane-tazobactam-nonsusceptible P. aeruginosa isolates were susceptible to ceftazidime-avibactam.

Cefiderocol MICs for meropenem-nonsusceptible (Fig. 1), ceftazidime-avibactam-nonsusceptible (Fig. 2), and ceftolozane-tazobactam-nonsusceptible (Fig. 3) isolates of P. aeruginosa (both North American and European isolates combined) demonstrated a rightward shift (of 1 doubling dilution) to higher cefiderocol MICs compared to each respective antimicrobial-susceptible subset; however, almost every nonsusceptible isolate (99.8 to 100%) remained susceptible to cefiderocol, with a MIC of ≤4 μg/mL.

The MIC_50_ and MIC_90_ of cefiderocol for isolates of A. baumannii complex from both North America and Europe were 0.12 and 1 μg/mL; 96.0% of isolates demonstrated cefiderocol MICs of ≤4 μg/mL (Table 1). Cefiderocol MIC distributions for meropenem-nonsusceptible isolates demonstrated approximately a 1-doubling-dilution rightward shift to higher MICs relative to the meropenem-susceptible isolate subset (Fig. 1); 94.2% of 2,810 isolates of meropenem-nonsusceptible A. baumannii complex remained cefiderocol susceptible, with a MIC_90_ value of 2 μg/mL. Ceftazidime-avibactam, ceftolozane-tazobactam, cefepime, and ciprofloxacin were inactive against A. baumannii complex. The MIC_90_ for colistin against meropenem-nonsusceptible A. baumannii complex was >8 μg/mL, and 12.8% of isolates were colistin resistant. Cefiderocol also inhibited 98.6% of S. maltophilia isolates at ≤1 μg/mL (Table 1). B. cepacia complex isolates tested with cefiderocol MIC_50_ and MIC_90_ values of ≤0.03 and 0.5 μg/mL within 1 doubling-dilution of the MIC_50_ (≤0.03 μg/mL) and MIC_90_ values (1 μg/mL) for the meropenem-nonsusceptible subset of isolates (Table 1).

Annual cefiderocol percent susceptible rates for isolates of *Enterobacterales* from North America (99.6 to 100% susceptible) and Europe (99.3 to 99.9% susceptible) varied over very narrow ranges (0.4 to 0.6%) (Table 2). Even less variation (0.1 to 0.2%) in annual cefiderocol percent susceptible rates was observed for P. aeruginosa. Annual cefiderocol percent susceptible rates for isolates of P. aeruginosa from North America ranged from 99.8% to 100%, and those for isolates from Europe ranged from 99.9% to 100%. Annual percent susceptible rates for A. baumannii demonstrated sporadic, nondirectional differences. The annual cefiderocol percent susceptible rate range was narrower for isolates of A. baumannii from North America (97.5 to 100%) than for isolates from Europe (90.4 to 97.5%). In total, there were 171 isolates of A. baumannii with cefiderocol MICs of ≥8 μg/mL (nonsusceptible) collected in Europe from 2014 to 2019. Of these isolates, 74.3% (127/171) were from one country (Russia); 127/437 (29.1%) of isolates from Russia were cefiderocol nonsusceptible, with annual rates of 28.2% (11/39) in 2014, 41.2% (7/17) in 2015, 24.1% (19/79) in 2016, 42.7% (50/117) in 2017, 31.9% (36/113) in 2018, and 5.6% (4/72) in 2019. Other European countries contributing >10 isolates over the study period submitted isolates with cefiderocol-nonsusceptible MICs at rates ranging from zero (no cefiderocol-nonsusceptible isolates) to 7.3% (8/109 isolates [United Kingdom]). Annual cefiderocol MIC distributions for *Enterobacterales*, P. aeruginosa, A. baumannii complex, S. maltophilia, and B. cepacia complex are provided in Tables S2 to S6 in the supplemental material.

**TABLE 2:** Annual antimicrobial susceptibility testing results for surveillance study isolates of *Enterobacterales*, P. aeruginosa, A. baumannii complex, and S. maltophilia collected in North America and Europe from 2014 to 2019 with MICs interpreted by CLSI criteria

Organism/location	Antimicrobial agent	CLSI MIC interpretation (% susceptible/intermediate/resistant)^*a*^						Maximum difference in annual % susceptible from 2014 to 2019
		2014	2015	2016	2017	2018	2019	
*Enterobacterales*								
North America, no. of isolates^*b*^		1,310	2,070	3,430	2,492	2,608	2,970	
	Cefiderocol	100/0/0	>99.9/0.1/0	99.8/0.1/0.1	100/0/0	99.9/0.1/0	99.6/0.4/0.1	0.4
	Cefepime	94.6/2.3/3.1	93.9/1.9/4.2	90.8/2.6/6.6	91.1/2.9/6.0	90.3/3.1/6.6	90.4/2.5/7.1	4.3
	Ceftazidime-avibactam	99.9/NA/0.1	100/NA/0.1	99.9/NA/0.1	99.9/NA/0.1	99.7/NA/0.3	99.7/NA/0.3	0.3
	Ceftolozane-tazobactam	94.2/1.9/3.9	94.9/1.6/3.6	94.5/1.4/4.1	93.8/1.9/4.3	94.4/1.8/3.7	94.3/1.5/4.3	1.1
	Ciprofloxacin	80.5/3.2/16.3	80.1/3.1/16.9	77.2/3.2/19.6	78.3/3/18.7	79.0/3.1/17.9	80.2/2.9/16.9	3.3
	Colistin	NA/97.5/2.5	NA/98.3/1.7	NA/97.3/2.7	NA/96.4/3.6	NA/97.4/2.6	NA/97.4/2.7	NA
	Meropenem	99.4/0.1/0.5	98.9/0.1/0.9	98.3/0.2/1.6	98.4/0.2/1.4	98.5/0.4/1.2	98.4/0.2/1.4	1.1

Europe, no. of isolates^*c*^		1,084	2,574	4,585	3,310	2,340	3,123	
	Cefiderocol	99.9/0.1/0	99.7/0.3/0	99.9/0.1/0	99.9/0.1/0	99.6/0.2/0.2	99.3/0.4/0.3	0.6
	Cefepime	87.6/2.5/10	81.8/3.0/15.2	81.7/3.3/15	80.3/3.1/16.7	79/4.2/16.8	79.2/3.4/17.4	8.4
	Ceftazidime-avibactam	98.6/NA/1.4	98.8/NA/1.2	98.8/NA/1.2	98.7/NA/1.3	98.4/NA/1.6	98.2/NA/1.8	0.6
	Ceftolozane-tazobactam	87.3/3.5/9.2	89.2/1.6/9.3	89.3/1.9/8.8	89.5/2.1/8.4	90.6/1.1/8.3	89.1/2.0/8.9	3.3
	Ciprofloxacin	78.7/3.1/18.2	71.7/3.3/25.0	70.8/2.9/26.4	69.5/3.4/27.0	69.7/3.9/26.4	68.5/3.3/28.2	10.2
	Colistin	NA/97.2/2.8	NA/96.5/3.5	NA/95.8/4.2	NA/95.5/4.5	NA/97.5/2.6	NA/97.3/2.7	NA
	Meropenem	96.5/0.3/3.2	95.9/0.5/3.7	95.3/0.5/4.1	95.2/0.7/4.1	95.0/0.3/4.8	94.5/0.5/5.0	2.0


P. aeruginosa								
North America, no. of isolates		253	512	798	630	644	711	
	Cefiderocol	100/0/0	99.8/0.2/0	100/0/0	100/0/0	100/0/0	99.9/0.1/0	0.2
	Cefepime	88.5/8.3/3.2	84.0/8.0/8.0	84.2/9.9/5.9	84.6/8.9/6.5	85.9/7.9/6.2	86.1/8.6/5.3	4.5
	Ceftazidime-avibactam	99.6/NA/0.4	97.3 NA/2.7	97.1/NA/2.9	97.1/NA/2.9	96.0/NA/4.0	96.9/NA/3.1	3.6
	Ceftolozane-tazobactam	99.6/0/0.4	96.7/1.8/1.6	98.1/0.9/1	97.8/1.3/1	97.7/0.8/1.6	97.8/0.4/1.8	2.9
	Ciprofloxacin	68.4/11.5/20.2	69.0/8/23.1	71.2/6/22.8	69.5/8.4/22.1	76.4/5.3/18.3	77.1/5.9/17	8.7
	Colistin	NA/98.8/1.2	NA/99.8/0.2	NA/99.9/0.1	NA/99.5/0.5	NA/99.8/0.2	NA/99.9/0.1	NA
	Meropenem	84.6/5.9/9.5	78.1/6.1/15.8	80.5/6.1/13.4	80.6/6.2/13.2	82.9/5.4/11.7	79.9/5.3/14.8	6.5

Europe, no. of isolates		218	580	1,066	911	601	776	
	Cefiderocol	100/0/0	100/0/0	99.9/0.1/0	100/0/0	100/0/0	99.9/0.1/0	0.1
	Cefepime	82.1/10.1/7.8	82.4/6.2/11.4	79.1/11.4/9.5	80.7/9.1/10.2	81.7/8.2/10.2	81.4/10.4/8.1	3.3
	Ceftazidime-avibactam	93.1/NA/6.9	91.2/NA/8.8	90.3/NA/9.7	90.8/NA/9.2	90.7/NA/9.3	91.8/NA/8.3	2.8
	Ceftolozane-tazobactam	91.3/2.3/6.4	90.7/1.9/7.4	90.2/1.1/8.6	90.6/0.7/8.8	90.7/0.8/8.5	91.5/0.9/7.6	1.3
	Ciprofloxacin	72.5/5.5/22	65.5/7.1/27.4	66.6/6/27.4	68.0/7.9/24.2	72.4/3.8/23.8	74.4/5.3/20.4	8.9
	Colistin	NA/99.1/0.9	NA/98.6/1.4	NA/99.3/0.8	NA/98.2/1.8	NA/99.2/0.8	NA/99.2/0.8	NA
	Meropenem	79.8/5.1/15.1	71.2/5.5/23.3	72.2/6.2/21.6	75.5/6.0/18.4	75.5/5.0/19.5	74.2/6.2/19.6	8.6
A. baumannii complex^*d*^								
North America, no. of isolates		158	162	422	363	452	442	
	Cefiderocol	100/0/0	98.2/1.9/0	97.6/1.7/0.7	97.5/0/2.5	99.1/0/0.9	97.7/1.8/0.5	2.5
	Cefepime	48.7/22.2/29.1	51.2/17.9/30.9	60.4/11.4/28.2	59.8/11.0/29.2	71.2/11.3/17.5	96.4/0.7/2.9	47.7
	Ciprofloxacin	36.1/0/63.9	35.2/0.6/64.2	57.4/1.7/41.0	55.9/1.7/42.4	71.9/1.6/26.6	66.3/1.1/32.6	36.7
	Colistin	NA/94.3/5.7	NA/95.7/4.3	NA/96.5/3.6	NA/96.4/3.6	NA/98.2/1.8	NA/98.9/1.1	NA
	Meropenem	43.7/1.9/54.4	46.3/1.2/52.5	63.3/0.5/36.3	65.6/1.4/33.1	75.9/1.8/22.4	69.9/2.0/28.1	32.2

Europe, no. of isolates		332	527	713	564	491	599	
	Cefiderocol	96.4/3.3/0.3	97.5/1.5/1.0	95.9/1.4/2.7	90.8/1.6/7.6	90.4/0.6/9.0	97.0/1.2/1.8	7.1
	Cefepime	22.0/14.8/63.3	30.0/11.4/58.6	29.5/12.6/57.9	32.5/8.5/59.0	43.4/7.3/49.3	83.8/1.2/15.0	61.8
	Ciprofloxacin	16.9/0.3/82.8	26.0/0/74.0	25.4/0.3/74.3	28.0/0.5/71.5	40.9/0.6/58.5	30.1/0.5/69.5	24.1
	Colistin	NA/87.1/13.0	NA/87.9/12.1	NA/84.6/15.4	NA/94.3/5.7	NA/94.3/5.7	NA/92.0/8.0	NA
	Meropenem	25.9/0.6/73.5	31.7/0.6/67.7	33.9/1.0/65.1	34.9/0.9/64.2	46.2/2.9/50.9	32.6/1.3/66.1	20.3


S. maltophilia ^*e*^								
North America, no. of isolates		21	140	200	187	198	217	
	Cefiderocol	100/NA/NA	96.4/NA/NA	99.0/NA/NA	100/NA/NA	98.5/NA/NA	99.5/NA/NA	3.6

Europe, no. of isolates		114	172	209	133	190	249	
	Cefiderocol	95.6/NA/NA	100/NA/NA	99.0/NA/NA	99.2/NA/NA	96.8/NA/NA	98.4/NA/NA	4.4

a Cefiderocol MICs were interpreted by CLSI breakpoints. CLSI MIC breakpoints for cefiderocol tested against *Enterobacterales* are as follows: susceptible, ≤4 μg/mL; intermediate, 8 μg/mL; and resistant, ≥16 μg/mL. CLSI MIC breakpoints for cefiderocol tested against P. aeruginosa are as follows: susceptible, ≤4 μg/mL; intermediate, 8 μg/mL; and resistant, ≥16 μg/mL. CLSI MIC breakpoints for cefiderocol tested against Acinetobacter spp. are as follows: susceptible, ≤4 μg/mL; intermediate, 8 μg/mL; and resistant, ≥16 μg/mL. CLSI MIC breakpoints for cefiderocol tested against S. maltophilia are as follows: susceptible, ≤1 μg/mL; and nonsusceptible, >1 μg/mL. For cefepime tested against *Enterobacterales* with MICs interpreted using CLSI breakpoints, susceptible dose-dependent isolates were classified as intermediate. NA, not available because MIC interpretative criteria are currently not available for this organism-antimicrobial agent combination.

b For *Enterobacterales* from North America tested against colistin, isolates with intrinsic resistance to colistin (Proteus spp., *Providencia* spp., Morganella morganii, and Serratia marcescens) were excluded from calculating CLSI MIC interpretation results for isolates from 2014 (*n *= 922), 2015 (*n *= 1,612), 2016 (*n *= 2,589), 2017 (*n *= 1,983), 2018 (*n *= 2,052), and 2019 (*n *= 2,078).

c For *Enterobacterales* from Europe tested against colistin, isolates with intrinsic resistance to colistin (Proteus spp., *Providencia* spp., Morganella morganii, and Serratia marcescens) were excluded from calculating CLSI MIC interpretation results for isolates from 2014 (*n *= 844), 2015 (*n *= 1,849), 2016 (*n *= 3,556), 2017 (*n *= 2,752), 2018 (*n *= 1,884), and 2019 (*n *= 2,267).

d CLSI (M100) does not publish MIC breakpoints for Acinetobacter spp. tested against ceftazidime-avibactam or ceftolozane-tazobactam.

e CLSI (M100) does not publish MIC breakpoints for S. maltophilia tested against cefepime, ceftazidime-avibactam, ceftolozane-tazobactam, ciprofloxacin, colistin, or meropenem.

Annual percent susceptible rates for ceftazidime-avibactam for isolates of *Enterobacterales* from North America (99.7 to 100%) and Europe (98.2 to 98.8%) were similar (<2% annual variation), while annual percent susceptible rates for ceftolozane-tazobactam were higher in isolates from North America (93.8 to 94.9%) than in those from Europe (87.3 to 90.6%) (Table 2). Annual percent susceptible rates for isolates of P. aeruginosa from North America were higher for both ceftazidime-avibactam (96.0 to 99.6%) and ceftolozane-tazobactam (96.7 to 99.6%) than for isolates from Europe (ceftazidime-avibactam, 90.3 to 93.1%; ceftolozane-tazobactam, 90.2 to 91.5%).

Isolates of *Enterobacterales*, P. aeruginosa, A. baumannii complex, S. maltophilia, and B. cepacia complex collected in 2019 were also tested against meropenem-vaborbactam and imipenem-relebactam (see Table S7 in the supplemental material). Meropenem-vaborbactam demonstrated *in vitro* activity similar to that of ceftazidime-avibactam against *Enterobacterales* (98.9% of isolates susceptible); <70% of meropenem-nonsusceptible *Enterobacterales* isolates were susceptible to meropenem-vaborbactam and imipenem-relebactam, compared to 93.2% susceptible for cefiderocol. Imipenem-relebactam was less active (83.9% susceptible) than ceftazidime-avibactam against P. aeruginosa, compared to 99.9% susceptible for cefiderocol. Meropenem-vaborbactam and imipenem-relebactam were largely inactive *in vitro* against clinical isolates of A. baumannii complex (MIC_90_, >16 μg/mL) and S. maltophilia (MIC_90_, >16 μg/mL).

## DISCUSSION

Data in the current study clearly demonstrate that the large majority of isolates of *Enterobacterales* (99.8%), P. aeruginosa (99.9%), A. baumannii complex (96.0%), and S. maltophilia (98.6%) collected across North America and Europe from 2104 to 2019 were susceptible to cefiderocol. Data in the current study confirm and expand upon data presented in earlier studies. Cefiderocol was previously reported to demonstrate potent *in vitro* activity against key Gram-negative pathogens (*Enterobacterales*, P. aeruginosa, Acinetobacter, *Stenotrophomonas*, and *Burkholderia*) but only limited activity against Gram-positive and anaerobic bacteria (7, 10, 15). International and regional surveillance studies (10, 15, 20–23) and resistant isolate collection profiling studies (8, 9, 11–14, 24) have reported ≥99% of *Enterobacterales*, P. aeruginosa, and S. maltophilia isolates and ≥96% of A. baumannii complex isolates have cefiderocol MICs of ≤4 μg/mL (10, 15, 20–23). Cefiderocol MICs were also ≤4 μg/mL for most carbapenem-resistant *Enterobacterales* (≥95% of isolates), P. aeruginosa (≥97%), and A. baumannii complex (≥91%) isolates, as well as MDR *Enterobacterales* (≥97%), P. aeruginosa (≥97%), and A. baumannii complex (≥90%) isolates (8–10, 14, 15, 20–24). Cefiderocol has dependably shown *in vitro* potency superior to those of ceftazidime-avibactam, ceftolozane-tazobactam, cefepime, ciprofloxacin, and colistin against clinical isolates of meropenem-resistant *Enterobacterales*, P. aeruginosa, and A. baumannii complex and to inhibit almost all isolates of *Enterobacterales* (>98%) and P. aeruginosa (>99%), with ceftazidime-avibactam-, ceftolozane-tazobactam-, cefepime-, ciprofloxacin- and colistin-resistant phenotypes at MICs of ≤4 μg/mL (8–10, 15, 20, 21, 24). Importantly, there was no appreciable cross-resistance between cefiderocol and ceftazidime-avibactam, ceftolozane-tazobactam, meropenem, or cefepime for *Enterobacterales* or P. aeruginosa, even though all are β-lactams. Most isolates resistant to newer β-lactam/β-lactamase inhibitor combinations remain susceptible to cefiderocol. In the current study, 91.6% of isolates of ceftazidime-avibactam-nonsusceptible *Enterobacterales* and 97.7% of isolates of ceftolozane-tazobactam-nonsusceptible *Enterobacterales* were susceptible to cefiderocol, as were 100% of isolates of ceftazidime-avibactam-nonsusceptible P. aeruginosa and 99.8% of isolates of ceftolozane-tazobactam-nonsusceptible P. aeruginosa. The current study also found that 93.9% of all isolates of B. cepacia complex had cefiderocol MICs of ≤1 μg/mL, and 95.5% of isolates had MICs of ≤4 μg/mL, similar to previous reports (10, 15, 20). We also confirmed that ceftazidime-avibactam and ceftolozane-tazobactam are largely inactive *in vitro* against clinical isolates of A. baumannii complex (MIC_90_, >64 μg/mL), S. maltophilia (MIC_90_, 64 to >64 μg/mL), and B. cepacia complex (MIC_90_, 8 to 32 μg/mL).

Single, specific mechanisms conferring resistance to cefiderocol in *Enterobacterales*, P. aeruginosa, and A. baumannii have not been identified, although the addition of avibactam, a β-lactamase inhibitor, to cefiderocol has been shown to lower the MICs for some cefiderocol-resistant isolates, primarily A. baumannii possessing various ESBLs. (15, 25, 26). In addition, in some isolates of Gram-negative bacilli with cefiderocol MICs ranging from 2 to 256 μg/mL, the addition of a β-lactamase inhibitor (e.g., clavulanic acid, avibactam, or dipicolinic acid) was shown to lower cefiderocol MICs (4). Cross-resistance between cefiderocol and other antibacterial classes has not been identified; generally, isolates of Gram-negative bacilli resistant to other antibacterial agents are reliably susceptible to cefiderocol (10, 15, 20). The frequency of resistance development in Gram-negative bacteria, including carbapenemase producers exposed to cefiderocol at 10 times the MIC, ranged from 10^−6^ to 10^−8^ (7, 27). Mutations in the upstream region of *pvdS* and *fecI* in P. aeruginosa, which could affect the expression of ferric siderophore uptake-related genes, were reported to increase cefiderocol MICs by 32-fold (7). Overproduction of AmpC, modifications of PBPs, and loss of the TonB energy-transducing protein or the siderophore receptors CirA and Fiu (*Enterobacterales*) or PiuA (not PirA) (P. aeruginosa) can also elevate cefiderocol MICs (4, 8). Cefiderocol does not induce AmpC β-lactamase production in P. aeruginosa and E. cloacae (28).

Even though resistance to cefiderocol has not been observed to be consistently mediated by the presence of specific carbapenemases (14), higher cefiderocol MICs among NDM-positive and PER-positive Gram-negative bacilli than among isolates producing other carbapenemases has been observed (9, 11, 14, 15). However, many isolates of NDM-producing *Enterobacterales* demonstrated cefiderocol MICs of ≤4 μg/mL (9, 11, 14, 15), and infections with NDM-producing *Enterobacterales* have been treated effectively with cefiderocol, as observed in clinical trials (29).

Different MIC breakpoints for cefiderocol have been published (16–19). Determining the *in vitro* susceptibility of clinical isolates to cefiderocol would benefit from the application of a uniform set of MIC and/or disk diffusion breakpoints. With the recent CLSI approval of clinical breakpoints for cefiderocol (17), CLSI and FDA MIC breakpoints are the same for *Enterobacterales* but not for P. aeruginosa (susceptible, ≤1 μg/mL) or Acinetobacter spp. (susceptible, ≤1 μg/mL), and the FDA has not published breakpoints for S. maltophilia (18). Cefiderocol breakpoints published by EUCAST are also different from those of the CLSI or FDA. Current EUCAST MIC breakpoints for *Enterobacterales* and P. aeruginosa are susceptible at ≤2 μg/mL and resistant at >2 μg/mL; non-species-related pharmacokinetic/pharmacodynamic MIC breakpoints for cefiderocol are also susceptible at ≤2 μg/mL and resistant at >2 μg/mL (19). Clearly, nonharmonized breakpoint criteria create perceived differences in susceptibility to cefiderocol, and to other agents, when MICs are interpreted by different MIC breakpoints. Depending upon the interpretive criteria used, isolates of P. aeruginosa with cefiderocol MICs of 2 or 4 μg/mL, for example, may be reported as susceptible, intermediate, or resistant. This is of particular importance for an agent such as cefiderocol because it is intended to be used against Gram-negative pathogens that have elevated MICs for most or all other potential therapeutic agents available.

We conclude that most current (2014 to 2019) clinical isolates of *Enterobacterales* (99.8%), P. aeruginosa (99.9%), A. baumannii complex (96.0%), and S. maltophilia (98.6%) in North America and Europe are susceptible to cefiderocol by the recently approved CLSI MIC breakpoints (17). Importantly, differences in the annual rates of percent susceptible for cefiderocol from 2014 to 2019 for isolates of *Enterobacterales* (North America range, 99.6 to 100% susceptible/year; Europe range, 99.3 to 99.9%) and P. aeruginosa (North America range, 99.8 to 100%; Europe range, 99.9 to 100%) were negligible. Annual percent susceptible rates for A. baumannii complex demonstrated sporadic, nondirectional differences (North America range, 97.5 to 100%; Europe range, 90.4 to 97.5%), primarily due to isolates from Russia. Annual percent susceptible rates for S. maltophilia also showed minor, nondirectional fluctuation (North America range, 96.4 to 100%; Europe range, 95.6 to 100%). *In vitro* susceptibility testing of cefiderocol may be of benefit when cefiderocol is being considered for treatment of patients infected with carbapenem-nonsusceptible, ceftazidime-avibactam-nonsusceptible, or ceftolozane-tazobactam-nonsusceptible isolates of *Enterobacterales* and P. aeruginosa, carbapenem-nonsusceptible isolates of A. baumannii complex, and MDR isolates of S. maltophilia.

## MATERIALS AND METHODS

### Bacterial isolates.

SIDERO-WT surveillance studies, sponsored by Shionogi & Co., Ltd., (Osaka, Japan), were run annually from November 2014 to December 2019. In those studies, predefined quotas of isolates of specific Gram-negative bacilli cultured from patients with intra-abdominal, urinary tract, lower respiratory tract, skin and soft tissue, or bloodstream infections were collected from clinical laboratories in North America and Europe as previously described (10, 15, 20). Tables S8 and S9 in the supplemental material summarize demographic data associated with the isolates collected in North America and Europe. In total, 31,896 isolates of *Enterobacterales*, 7,700 isolates of P. aeruginosa, 5,225 isolates of A. baumannii complex, 2,030 isolates of S. maltophilia, and 425 isolates of B. cepacia complex were collected in North America and Europe from 2014 to 2019. All isolates were shipped to IHMA (Schaumburg, IL, USA), where their identities were confirmed using matrix-assisted laser desorption ionization–time of flight mass (MALDI-TOF) mass spectrometry (Bruker Daltonics, Billerica, MA, USA).

### Antimicrobial susceptibility testing.

CLSI-defined broth microdilution susceptibility testing was performed at IHMA using custom in-house-prepared broth microdilution panels (10, 16, 30). Cefiderocol was tested in Chelex-treated iron-depleted cation-adjusted Mueller-Hinton broth; all other antimicrobial agents were tested in standard CAMHB (BBL, Becton Dickinson, Sparks, MD) (16). MIC endpoints for each agent tested were read and interpreted using CLSI standards (16). Cefiderocol MICs were interpreted using CLSI-approved (February 2021) MIC breakpoints for *Enterobacterales*, P. aeruginosa, and Acinetobacter species of ≤4 μg/mL for susceptible, 8 μg/mL for intermediate, and ≥16 μg/mL for resistant and MIC breakpoints for S. maltophilia of ≤1 μg/mL for susceptible and >1 μg/mL for nonsusceptible (17).
