# Supplementary material for: In Vitro Susceptibility of Gram-Negative Pathogens to Cefiderocol in Five Consecutive Annual Multinational SIDERO-WT Surveillance Studies, 2014 to 2019
Source: Antimicrob Agents Chemother. 2022 Feb 15;66(2):e01990-21. doi: 10.1128/AAC.01990-21 (PMC8846469; doi:10.1128/AAC.01990-21)
Supplement: Supplemental file 1 — Supplemental tables. Download AAC.01990-21-s0001.pdf, PDF file, 0.3 MB [file aac.01990-21-s0001.pdf]

SUPPLEMENTARY DATA

TABLE S1 Cefiderocol MICs interpreted by approved CLSI, FDA, and EUCAST MIC breakpoints for combined North America and Europe isolates of *Enterobacterales*, *P. aeruginosa*, *A. baumannii* complex, and *S. maltophilia* collected in five consecutive annual SIDERO-WT surveillance studies from 2014 to 2019

Organism/phenotype (no. of isolates)	CLSI MIC interpretation[a]			FDA MIC interpretation[b]			EUCAST MIC interpretation[c]	
	% Susceptible	% Intermediate	% Resistant	% Susceptible	% Intermediate	% Resistant	% Susceptible	% Resistant
Enterobacterales								
All isolates (31,896)	99.8	0.2	0.1	99.8	0.2	0.1	98.3	1.7
Meropenem-nonsusceptible (1,021)	96.7	2.5	0.8	96.7	2.5	0.8	79.9	20.1
P. aeruginosa								
All isolates (7,700)	99.9	0.1	0	97.5	1.9	0.6	99.4	0.6
Meropenem-nonsusceptible (1,759)	99.8	0.2	0	94.2	4.3	1.5	98.5	1.5
A. baumannii complex								
All isolates (5,225)	96.0	1.3	2.7	90.0	4.1	5.9	94.1	5.9
Meropenem-nonsusceptible (2,810)	94.2	2.1	3.7	84.9	6.2	8.9	91.1	8.9
S. maltophilia								
All isolates (2,030)	98.6	NA	NA	NA	NA	NA	99.6	0.4

[a] CLSI approved MIC clinical breakpoints for *Enterobacterales*, *P. aeruginosa*, and *Acinetobacter* spp. are ≤4 µg/ml (susceptible), 8 µg/ml (intermediate), and ≥16 µg/ml (resistant), and for *S. maltophilia* are ≤1 µg/ml (susceptible) and >1 µg/ml (nonsusceptible). These MIC clinical breakpoints were approved by the CLSI in February 2021 (https://clsi.org/meetings/ast-file-resources/).

[b] FDA approved MIC breakpoints for *Enterobacterales* are ≤4 µg/ml (susceptible), 8 µg/ml (intermediate), and ≥16 µg/ml (resistant); and for *P. aeruginosa* and *Acinetobacter* spp. are ≤1 µg/ml (susceptible), 2 µg/ml (intermediate), and ≥4 µg/ml (resistant) (https://www.fda.gov/drugs/development-resources/fda-recognized-antimicrobial-susceptibility-test-interpretative-criteria). The FDA has not published cefiderocol MIC breakpoints for *S. maltophilia*.

[c] EUCAST (breakpoint tables v. 11.0) MIC clinical breakpoints for cefiderocol for *Enterobacterales* and *P. aeruginosa* are susceptible (≤2 µg/ml) and resistant (>2 µg/ml) (http://www.eucast.org/clinical_breakpoints/). Identical susceptible and resistant non-species related pharmacokinetic/pharmacodynamic MIC breakpoints for cefiderocol are also published by EUCAST which were applied to *Acinetobacter* spp. and *S. maltophilia* MIC data.

TABLE S2 Cefiderocol MIC distributions for *Enterobacterales* by year from 2014 to 2019

Year		MIC (µg/ml)										
		≤0.03	0.06	0.12	0.25	0.5	1	2	4	8	≥16	Total
2014	No. of isolates	721	378	512	395	202	116	48	21	1	0	2394
	% of isolates	30.1	15.8	21.4	16.5	8.4	4.8	2.0	0.9	0.1	0	100
2015	No. of isolates	1500	842	847	691	382	222	113	38	8	1	4644
	% of isolates	32.3	18.1	18.2	14.9	8.2	4.8	2.4	0.8	0.2	0.1	100
2016	No. of isolates	2396	956	1211	1428	1077	602	242	92	6	5	8015
	% of isolates	29.9	11.9	15.1	17.8	13.4	7.5	3.0	1.2	0.1	0.1	100
2017	No. of isolates	1869	614	767	1178	688	417	151	112	4	2	5802
	% of isolates	32.2	10.6	13.2	20.3	11.9	7.2	2.6	1.9	0.1	0.03	100
2018	No. of isolates	1635	566	682	830	707	314	134	67	8	5	4948
	% of isolates	33.0	11.4	13.8	16.8	14.3	6.3	2.7	1.4	0.2	0.1	100
2019	No. of isolates	1426	890	1121	1105	771	385	220	141	24	10	6093
	% of isolates	23.4	14.6	18.4	18.1	12.7	6.3	3.6	2.3	0.4	0.2	100
Total	No. of isolates	9547	4246	5140	5627	3827	2056	908	471	51	23	31896
	% of isolates	29.9	13.3	16.1	17.6	12.0	6.5	2.8	1.5	0.2	0.1	100

TABLE S3 Cefiderocol MIC distributions for *P. aeruginosa* by year from 2014 to 2019

Year		MIC (µg/ml)										
		≤0.03	0.06	0.12	0.25	0.5	1	2	4	8	≥16	Total
2014	No. of isolates	111	96	117	82	42	17	6	0	0	0	471
	% of isolates	23.6	20.4	24.8	17.4	8.9	3.6	1.3	0	0	0	100
2015	No. of isolates	229	280	247	188	75	52	14	6	1	0	1092
	% of isolates	21.0	25.6	22.6	17.2	6.9	4.8	1.3	0.5	0.1	0	100
2016	No. of isolates	194	334	591	461	167	72	36	8	1	0	1864
	% of isolates	10.4	17.9	31.7	24.7	9.0	3.9	1.9	0.4	0.1	0	100
2017	No. of isolates	162	199	427	462	166	80	31	14	0	0	1541
	% of isolates	10.5	12.9	27.7	30.0	10.8	5.2	2.0	0.9	0	0	100
2018	No. of isolates	100	152	343	392	166	60	29	3	0	0	1245
	% of isolates	8.0	12.2	27.6	31.5	13.3	4.8	2.3	0.2	0	0	100
2019	No. of isolates	129	228	406	408	209	65	31	9	2	0	1487
	% of isolates	8.7	15.3	27.3	27.4	14.1	4.4	2.1	0.6	0.1	0	100
Total	No. of isolates	925	1289	2131	1993	825	346	147	40	4	0	7700
	% of isolates	12.0	16.7	27.7	25.9	10.7	4.5	1.9	0.5	0.1	0	100

TABLE S4 Cefiderocol MIC distributions for *A. baumannii* complex by year from 2014 to 2019

Year		MIC										Total
		≤0.03	0.06	0.12	0.25	0.5	1	2	4	8	≥16	
2014	No. of isolates	71	157	108	44	35	43	12	8	11	1	490
	% of isolates	14.6	32	22	9	7.1	8.8	2.5	1.6	2.2	0.2	100
2015	No. of isolates	141	180	159	74	47	59	9	4	11	5	689
	% of isolates	20.5	26.1	23.1	10.7	6.8	8.6	1.3	0.6	1.6	0.7	100
2016	No. of isolates	77	178	227	249	151	131	63	20	17	22	1135
	% of isolates	6.8	15.7	20	21.9	13.3	11.5	5.6	1.8	1.5	1.9	100
2017	No. of isolates	98	157	170	189	110	65	44	33	9	52	927
	% of isolates	10.6	16.9	18.3	20.4	11.9	7	4.7	3.6	1	5.6	100
2018	No. of isolates	93	186	213	166	109	74	32	19	3	48	943
	% of isolates	9.9	19.7	22.6	17.6	11.6	7.8	3.4	2	0.3	5.1	100
2019	No. of isolates	69	195	280	168	133	98	52	18	15	13	1041
	% of isolates	6.6	18.7	26.9	16.1	12.8	9.4	5	1.7	1.5	1.3	100
Total	No. of isolates	549	1053	1157	890	585	470	212	102	66	141	5225
	% of isolates	10.5	20.1	22.1	17	11.2	9	4.1	2	1.3	2.7	100

TABLE S5 Cefiderocol MIC distributions for *S. maltophilia* by year from 2014 to 2019

Year		MIC										Total
		≤0.03	0.06	0.12	0.25	0.5	1	2	4	8	≥16	
2014	No. of isolates	31	22	26	17	6	3	4	0	0	0	109
	% of isolates	28.4	20.2	23.9	15.6	5.5	2.8	3.7	0	0	0	100
2015	No. of isolates	128	73	60	50	13	8	4	2	0	0	338
	% of isolates	37.9	21.6	17.8	14.8	3.8	2.4	1.9	0.6	0	0	100
2016	No. of isolates	99	100	107	62	26	11	2	0	0	2	409
	% of isolates	24.2	24.4	26.2	15.2	6.4	2.7	0.5	0	0	0.5	100
2017	No. of isolates	139	60	54	45	13	8	1	0	0	0	320
	% of isolates	43.4	18.9	16.9	14.1	4.1	2.5	0.3	0	0	0	100
2018	No. of isolates	70	78	94	87	37	13	6	1	0	2	388
	% of isolates	18.0	20.1	24.2	22.4	9.5	3.4	1.5	0.3	0	0.5	100
2019	No. of isolates	186	145	77	29	17	7	4	1	0	0	466
	% of isolates	39.9	31.1	16.5	6.2	3.6	1.5	0.9	0.2	0	0	100
Total	No. of isolates	653	478	418	290	112	50	42	4	0	4	2,030
	% of isolates	32.2	23.5	20.6	14.3	5.5	2.5	2.1	0.2	0	0.2	100

TABLE S6 Cefiderocol MIC distributions for *B. cepacia* complex by year from 2014 to 2019

Year		MIC										Total
		≤0.03	0.06	0.12	0.25	0.5	1	2	4	8	≥16	
2014	No. of isolates	1	0	0	0	0	0	0	0	0	0	1
	% of isolates	100	0	0	0	0	0	0	0	0	0	100
2015	No. of isolates	10	4	0	0	1	1	0	0	0	1	18
	% of isolates	55.6	22.2	0	0	5.6	5.6	0	0	0	5.6	100
2016	No. of isolates	64	6	7	2	2	2	1	0	2	3	89
	% of isolates	71.9	6.7	7.9	2.2	2.2	2.2	1.1	0	2.2	3.4	100
2017	No. of isolates	53	3	3	1	0	0	0	0	0	2	62
	% of isolates	85.5	4.8	4.8	1.6	0	0	0	0	0	3.2	100
2018	No. of isolates	76	4	4	2	1	3	2	0	0	3	95
	% of isolates	80.0	4.2	4.2	2.1	1.1	3.2	2.1	0	0	3.2	100
2019	No. of isolates	104	14	17	3	5	5	1	3	2	6	160
	% of isolates	65.0	8.8	10.6	1.9	3.1	3.1	0.6	1.9	1.3	3.8	100
Total	No. of isolates	309	31	31	8	9	11	4	3	4	15	425
	% of isolates	72.7	7.3	7.3	1.9	2.1	2.6	0.9	0.7	0.9	3.5	100

TABLE S7. Cumulative antimicrobial susceptibility testing results for cefiderocol and selected comparators including meropenem-vaborbactam and imipenem-relebactam from SIDERO-WT surveillance study isolates of *Enterobacterales*, *P. aeruginosa*, *A. baumannii* complex, *S. maltophilia*, and *B. cepacia* complex collected in North America and Europe in 2019

Organism/phenotype (no. of isolates)	Antimicrobial agent	MIC (µg/ml)			CLSI MIC interpretation[a,b,c]		
		Range	MIC_{50}	MIC_{90}	% Susceptible	% Intermediate	% Resistant
Enterobacterales[d]							
All isolates (6,093)	Cefiderocol	≤0.03 - 16	0.12	1	99.4	0.4	0.2
	Cefepime	≤0.12 - >16	≤0.12	>16	84.7	3.0	12.4
	Ceftazidime-avibactam	≤0.12 - >16	≤0.12	0.5	98.9	NA	1.1
	Ceftolozane-tazobactam	≤0.12 - >16	0.25	2	91.6	1.7	6.7
	Meropenem-vaborbactam	≤0.06 - >16	≤0.06	0.12	98.9	0.2	1.0
	Imipenem-relebactam	≤0.03 - >16	0.25	2	85.0	9.5	5.5
	Meropenem	≤0.06 - >16	≤0.06	0.12	96.4	0.4	3.3
Meropenem-nonsusceptible (MIC ≥2 µg/ml) (220)	Cefiderocol	≤0.03 - 16	1	4	93.2	5.5	1.4
	Cefepime	≤0.12 - >16	>16	>16	7.3	5.9	86.8
	Ceftazidime-avibactam	≤0.12 - >16	1	>16	74.1	NA	25.9
	Ceftolozane-tazobactam	0.5 - >16	>16	>16	5.0	2.7	92.3
	Meropenem-vaborbactam	≤0.06 - >16	2	>16	68.2	4.6	27.3
	Imipenem-relebactam	0.06 - >16	1	>16	54.1	5.9	40.0
	Meropenem	2 - >16	>16	>16	0	10.0	90.0
Ceftazidime-avibactam-nonsusceptible (MIC ≥16 µg/ml) (65)	Cefiderocol	≤0.03 - 16	2	4	90.8	4.6	4.6
	Cefepime	2 - >16	>16	>16	4.6	6.2	89.2
	Ceftazidime-avibactam	16 - >16	>16	>16	0	NA	100
	Ceftolozane-tazobactam	0.5 - >16	>16	>16	1.5	0	98.5
	Meropenem-vaborbactam	≤0.06 - >16	>16	>16	32.3	9.2	58.5
	Imipenem-relebactam	0.25 - >16	16	>16	9.2	4.6	86.2
	Meropenem	≤0.06 - >16	>16	>16	12.3	4.6	83.1
Ceftolozane-tazobactam-nonsusceptible (MIC ≥4 µg/ml) (511)	Cefiderocol	≤0.03 - 16	1	4	94.7	4.1	1.2
	Cefepime	≤0.12 - >16	>16	>16	27.6	13.7	58.7
	Ceftazidime-avibactam	≤0.12 - >16	1	>16	87.5	NA	12.5
	Ceftolozane-tazobactam	4 - >16	>16	>16	0	20.7	79.3
	Meropenem-vaborbactam	≤0.06 - >16	≤0.06	16	86.5	2.0	11.6
	Imipenem-relebactam	≤0.03 - >16	0.25	8	76.9	5.3	17.8
	Meropenem	≤0.06 - >16	0.25	>16	59.1	3.3	37.6
P. aeruginosa							
All isolates (1,487)	Cefiderocol	≤0.03 - 8	0.12	0.5	99.9	0.1	0
	Cefepime	≤0.12 - >16	4	16	83.7	9.6	6.8
	Ceftazidime-avibactam	≤0.12 - >16	2	8	94.2	NA	5.8
	Ceftolozane-tazobactam	≤0.12 - >16	0.5	2	94.5	0.7	4.8
	Meropenem-vaborbactam	≤0.06 - >16	0.5	16	NA	NA	NA
	Imipenem-relebactam	≤0.03 - >16	0.5	2	83.9	7.1	9.0
	Meropenem	≤0.06 - >16	0.5	16	76.9	5.8	17.3
Meropenem-nonsusceptible (MIC ≥4 µg/ml) (343)	Cefiderocol	≤0.03 - 8	0.25	1	99.4	0.6	0

Organism/phenotype (no. of isolates)	Antimicrobial agent	MIC (µg/ml)			CLSI MIC interpretation[a,b,c]		
		Range	MIC_{50}	MIC_{90}	% Susceptible	% Intermediate	% Resistant
	Cefepime	0.5 - >16	8	>16	51.9	23.3	24.8
	Ceftazidime-avibactam	0.5 - >16	4	>16	77.0	NA	23.0
	Ceftolozane-tazobactam	0.25 - >16	1	>16	77.6	2.3	20.1
	Meropenem-vaborbactam	0.25 - >16	8	>16	NA	NA	NA
	Imipenem-relebactam	0.25 - >16	2	>16	32.9	28.3	38.8
	Meropenem	4 - >16	16	>16	0	25.1	74.9
Ceftazidime-avibactam-nonsusceptible (MIC ≥16 µg/ml) (86)	Cefiderocol	≤0.03 - 4	0.25	2	100	0	0
	Cefepime	8 - >16	>16	>16	2.3	19.8	77.9
	Ceftazidime-avibactam	16 - >16	>16	>16	0	NA	100
	Ceftolozane-tazobactam	1 - >16	>16	>16	31.4	4.7	64.0
	Meropenem-vaborbactam	0.5 - >16	>16	>16	NA	NA	NA
	Imipenem-relebactam	0.25 - >16	16	>16	11.6	14.0	74.4
	Meropenem	1 - >16	>16	>16	8.1	2.3	89.5
Ceftolozane-tazobactam-nonsusceptible (MIC ≥8 µg/ml) (82)	Cefiderocol	≤0.03 - 4	0.25	2	100	0	0
	Cefepime	4 - >16	>16	>16	6.1	23.2	70.7
	Ceftazidime-avibactam	1 - >16	>16	>16	28.1	NA	72.0
	Ceftolozane-tazobactam	8 - >16	>16	>16	0	12.2	87.8
	Meropenem-vaborbactam	0.25 - >16	>16	>16	NA	NA	NA
	Imipenem-relebactam	0.25 - >16	>16	>16	11.0	8.5	80.5
	Meropenem	0.5 - >16	>16	>16	6.1	2.4	91.5
A baumannii complex							
All isolates (1,041)	Cefiderocol	≤0.03 - >64	0.12	1	97.3	1.4	1.3
	Cefepime	≤0.12 - >16	0.25	16	89.2	1.0	9.9
	Meropenem-vaborbactam	≤0.06 - >16	2	>16	NA	NA	NA
	Imipenem-relebactam	≤0.03 - >16	0.5	>16	51.4	1.3	47.4
	Meropenem	≤0.06 - >16	4	>16	48.4	1.6	50.0
Meropenem-nonsusceptible (MIC ≥4 µg/ml) (537)	Cefiderocol	≤0.03 - >64	0.25	2	95.0	2.6	2.4
	Cefepime	≤0.12 - >16	0.5	>16	80.1	1.7	18.3
	Meropenem-vaborbactam	0.25 - >16	>16	>16	NA	NA	NA
	Imipenem-relebactam	0.25 - >16	>16	>16	5.8	2.4	91.8
	Meropenem	4 - >16	>16	>16	0	3.2	96.8
S. maltophilia							
All isolates (466)	Cefiderocol	≤0.03 - 4	0.06	0.25	100	0	0
	Cefepime	≤0.12 - >16	≤0.12	1	NA	NA	NA
	Meropenem-vaborbactam	≤0.06 - >16	>16	>16	NA	NA	NA
	Imipenem-relebactam	≤0.03 - >16	>16	>16	NA	NA	NA
	Meropenem	≤0.06 - >16	>16	>16	NA	NA	NA
B. cepacia complex							
All isolates (160)	Cefiderocol	≤0.03 - 32	≤0.03	1	NA	NA	NA
	Meropenem-vaborbactam	0.12 - >16	1	4	NA	NA	NA

Organism/phenotype (no. of isolates)	Antimicrobial agent	MIC (µg/ml)			CLSI MIC interpretation[a,b,c]		
		Range	MIC_{50}	MIC_{90}	% Susceptible	% Intermediate	% Resistant
	Imipenem-relebactam	0.06 - >16	1	4	NA	NA	NA
	Meropenem	0.5 - >16	8	>16	35.0	40.0	25.0
Meropenem-nonsusceptible (MIC ≥8 µg/ml) (104)	Cefiderocol	≤0.03 - 32	≤0.03	0.5	NA	NA	NA
	Meropenem-vaborbactam	0.5 - >16	1	4	NA	NA	NA
	Imipenem-relebactam	0.25 - >16	1	4	NA	NA	NA
	Meropenem	8 - >16	8	>16	0	61.5	38.5

[a] Cefiderocol MICs and MICs for other antimicrobial agents were interpreted by CLSI breakpoints. CLSI MIC breakpoints for cefiderocol tested against *Enterobacterales* are: susceptible, ≤4 µg/ml; intermediate, 8 µg/ml; and resistant, ≥16 µg/ml. CLSI MIC breakpoints for cefiderocol tested against *P. aeruginosa* are: susceptible, ≤4 µg/ml; intermediate, 8 µg/ml; and resistant, ≥16 µg/ml. CLSI MIC breakpoints for cefiderocol tested against *Acinetobacter* spp. are: susceptible, ≤4 µg/ml; intermediate, 8 µg/ml; and resistant, ≥16 µg/ml. CLSI MIC breakpoints for cefiderocol tested against *S. maltophilia* are: susceptible, ≤1 µg/ml; nonsusceptible, >1 µg/ml. CLSI currently does not publish cefiderocol MIC breakpoints for *B. cepacia* complex.

[b] For cefepime tested against *Enterobacterales* with MICs interpreted using CLSI breakpoints, susceptible-dose dependent isolates were classified as intermediate.

[c] NA, not available. MIC interpretative criteria are currently not available for this organism-antimicrobial agent combination.

TABLE S8 Demographic information associated with the 14,880 isolates of *Enterobacterales*, 3,548 isolates of *P. aeruginosa*, 1,999 isolates of *A. baumannii* complex, 963 isolates of *S. maltophilia*, and 196 isolates of *B. cepacia* complex tested by the SIDERO-WT surveillance program from North America from 2014 to 2019

		Enterobacterales (no. of isolates)	*P. aeruginosa* (no. of isolates)	*A. baumannii* complex (no. of isolates)	*S. maltophilia* (no. of isolates)	*B. cepacia* complex (no. of isolates)
Specimen source						
	Intra-abdominal	1,695	205	46	58	3
	Urinary tract	4,614	617	348	50	10
	Lower respiratory tract	3,727	1,739	817	671	129
	Skin and soft tissue	1,712	512	437	108	17
	Bloodstream	2,732	353	289	45	31
	Other	400	122	62	31	6
	Total	14,880	3,548	1,999	963	196
Country (number of participating medical centers)						
	Canada (11)	2,038	461	243	175	41
	United States (65)	12,842	3,087	1,756	788	155
	Total	14,880	3,548	1,999	963	196
Year of isolation						
	2014	1,310	253	158	21	0
	2015	2,070	512	162	140	10
	2016	3,430	798	422	200	41
	2017	2,492	630	363	187	35
	2018	2,608	644	452	198	46
	2019	2,970	711	442	217	64
	Total	14,880	3,548	1,999	963	196

TABLE S9 Demographic information associated with the 17,016 isolates of *Enterobacterales*, 4,152 isolates of *P. aeruginosa*, 3,266 isolates of *A. baumannii* complex, 1,067 isolates of *S. maltophilia*, and 229 isolates of *B. cepacia* complex tested by the SIDERO-WT surveillance program from Europe from 2014 to 2019

		Enterobacterales (no. of isolates)	*P. aeruginosa* (no. of isolates)	*A baumannii* complex (no. of isolates)	*S. maltophilia* (no. of isolates)	*B. cepacia* complex (no. of isolates)
Specimen source						
	Intra-abdominal	2,815	409	215	81	1
	Urinary tract	3,754	512	338	72	12
	Lower respiratory tract	4,213	1,882	1,531	638	179
	Skin and soft tissue	2,881	793	627	101	11
	Bloodstream	3,029	480	445	153	25
	Other	324	76	70	22	1
	Total	17,016	4,152	3,226	1,067	229
Country (number of participating medical centers)						
	Austria (3)	35	0	0	0	0
	Belgium (4)	83	0	0	0	0
	Croatia (1)	3	0	0	0	0
	Czech Republic (4)	1,350	334	213	72	39
	Denmark (3)	36	0	0	0	0
	France (11)	1,689	419	289	129	18
	Germany (14)	1,918	473	265	164	15
	Greece (6)	1,490	304	420	106	3
	Hungary (5)	1,114	278	210	64	11
	Italy (13)	1,947	463	505	154	24
	Latvia (1)	3	0	0	0	0
	Lithuania (2)	5	0	0	0	0
	Netherlands (2)	45	0	0	0	0
	Poland (3)	12	0	0	0	0
	Portugal (5)	86	0	0	0	0
	Romania (2)	11	0	0	0	0
	Russia (14)	1,735	520	437	101	18
	Serbia (1)	1	0	0	0	0
	Slovenia (1)	1	0	0	0	0
	Spain (14)	1,930	482	336	155	51
	Sweden (4)	581	124	10	8	1
	Switzerland (0)	0	0	0	0	0

	Turkey (8)	1,376	369	432	64	31
	United Kingdom (8)	1,565	386	109	114	18
	Total	17,016	4,152	3,226	1,067	229
Year of isolation						
	2014	1,084	218	332	114	1
	2015	2,574	580	527	172	8
	2016	4,585	1,066	713	209	48
	2017	3,310	911	564	133	27
	2018	2,340	601	491	190	49
	2019	3,123	776	599	249	96
	Total	17,016	4,152	3,226	1,067	229
